# Antimicrobial resistance and virulence determinants in coagulase-negative staphylococci isolated mainly from preterm neonates

**DOI:** 10.1371/journal.pone.0236713

**Published:** 2020-08-04

**Authors:** Aishah Al-Haqan, Samar S. Boswihi, Seema Pathan, Edet E. Udo

**Affiliations:** 1 Microbiology Laboratory, Maternity Hospital, Kuwait city, Kuwait; 2 Department of Microbiology, Faculty of Medicine, Kuwait University, Kuwait city, Kuwait; Cairo University, EGYPT

## Abstract

Coagulase-negative staphylococci (CoNS) are the most common isolates from blood culture in neonates resulting in high mortality and morbidity. This study investigated CoNS obtained from blood cultures of neonates for antibiotic resistance and virulence factors, and possible association with inflammatory response (C-reactive protein). A total of 93 CoNS isolates were collected from 76 blood cultures of neonates at the Maternity hospital in Kuwait in a six-month period and investigated for susceptibility to antibiotics, carriage of staphylococcal cassette chromosome *mec* (SCC*mec*), and virulence-associated genes. The 93 CoNS isolates consisted of *S*. *epidermidis* (76; 81.7%), *S*. *capitis* (12; 12.9%), *S*. *hominis* (2; 2.1%), *S*. *warneri* (2; 2.1%) and *S*. *haemolyticus* (1; 1.0%). Eighty-six (92.4%) of the isolates were resistant to cefoxitin (MR-CoNS) while 49 (52.7%) expressed multi-antibiotic resistance. The methicillin-resistant isolates (MR-CoNS) carried SCC*mec* III, SCC*mec* IVa and four combinations of SCC*mec* types including SCC*mec* types I+IVa (one *S*. *warneri* and 25 *S*. *epidermidis* isolates), types I+III (one *S*. *epidermidis* isolate), types III+IVa (six *S*. *epidermidis* isolates) and types I+III+IVa (one *S*. *epidermidis* isolate). The most common virulence-related genes were *icaC*, *seb*, *arc* detected in 69.7%, 60.5%, 40.8% of the isolates respectively. Two isolates were positive for *tst1*. No association between C-reactive protein and antibiotic resistance or virulence factors was established. This study revealed that *S*. *epidermidis* carrying different SCC*mec* genetic elements, was the dominant CoNS species isolated from neonatal blood cultures with 90.3% and 36.6% of the isolates positive for genes for biofilm and ACME production respectively.

## Introduction

Neonatal sepsis is a fatal infection that appears in the first 28 days of a neonate’s life or up to 4 weeks after the expected due date for preterm infants [1]. Several factors are associated with neonatal sepsis. These include prematurity, low birth weight and immature immune system [1]. Neonatal sepsis is characterized by abdominal distention, apnea, bradycardia, fever, hypothermia, feeding difficulties, lethargy and neutropenia [1, 2]. Neonatal sepsis is classified as either early-onset (0–3 day of life) or late-onset sepsis (4–90 days of life) [1, 2]. The early-onset sepsis occurs in infants that acquire bacteria from infected and non-infected mothers, while late-onset sepsis occurs in infants that acquire the bacterial pathogen either from the hospital environment or a healthcare worker [1, 3]. Although current prevention measures have reduced the incidence of mortality due to neonatal sepsis from >80% in 1940s to 20% in the late 1960s [4], neonates are still at risk of acquiring the infection because of their continual exposure to pathogens, particularly coagulase-negative staphylococci (CoNS) found in colonized parents, healthcare workers and healthcare environments [1, 3, 5].

*Staphylococcus epidermidis* is a frequent cause of late-onset sepsis [1, 6]. It can colonize the skin, nose and umbilical cord of neonates within days of their admission to the neonatal intensive care unit (NICU) [6]. Neonatal mortality due to *S*. *epidermidis* sepsis can be as high as 4.8% especially in neonates with very low birth weight [6].

The C-reactive protein (CRP) is a protein secreted by the human liver in response to inflammation due to an infection or any tissue injury [7] and is used as a laboratory marker to diagnose neonatal bacteraemia [7]. CRP value of ≥ 10 mg/L is the most commonly cut-off used to identify infection in neonates and it is best measured within 24 to 48 h of onset of infection [8]. The sensitivity of the CRP test can be affected by several factors including premature rupture of membranes, lower gestational age or lower birth body weight, and the causative pathogen isolated [7, 8]. Studies have revealed that patients suffering from CoNS bacteraemia have low CRP values [7, 9–11].

Studies have also shown that CoNS causing late onset sepsis in neonates are often multi-resistant to antibiotics [12]. Antibiotic resistance in CoNS, especially against beta-lactam antibiotics, has increased over the years [11, 12]. The *mecA* gene, that encodes methicillin resistance, has been reported in as much as 80% of the CoNS isolates causing late-onset sepsis [13]. The *mecA* gene is carried on a mobile genetic island named staphylococcal cassette chromosome *mec* (SCC*mec*). Eleven types of SCC*mec* (I-XI), have been identified with SCC*mec* types III and V shown to be more prevalent in methicillin-resistant-CoNS (MR-CoNS) [14].

In addition to antibiotic resistance, CoNS elaborate virulence factors including the ability to form biofilm which is considered as an important virulence determinant [15]. Sepsis caused by biofilm-forming strains of CoNS has been associated with decreased host inflammatory response due to the inability of the immune system to counteract the infection [15]. Biofilms also serves as protective physical barriers for CoNS by not allowing the antibiotics to diffuse into the host cells [15]. The ability of biofilms to adhere to inanimate objects such as indwelling medical devices is mostly facilitated by surface proteins known as the polysaccharide intercellular adhesin (PIA). These surface proteins are regulated by *icaADBC* genes that are organized in an operon known as *ica* operon [13]. It has been shown that the carriage of *icaA* and *mecA* allows CoNS to persist for longer periods of time over the susceptible species in the hospital environment [13].

The arginine catabolic mobile element (ACME) is another virulence factor that has been associated with CoNS. ACME is a genomic island that was recognized to contribute to the fitness and ability of the USA300 Community-associated MRSA to colonize the skin and mucous membranes [16]. The ACME has also been detected in a few other *S*. *aureus* lineages and CoNS species, such as, *S*. *haemolyticus*, *S*. *capitis and S*. *epidermidis* [17, 18]. The ACME gene consist of three gene clusters including *arcR/A/D/B/C* (arc operon), *opp3A/B/C/D/E* (opp3 operon) and *kdpE/D/A/B/C* (kdp operon) genes [16, 19]. The *arc* operon encodes arginine deaminase pathway, the opp3 operon encodes oligopeptide permease ABC transporter, while kdp encodes potassium ABC transporter [18]. Based on the carriage of the *arc*, *opp3* and *kdp* clusters, five ACME types have been identified [16, 19]. Type I contains both the *arc* and *opp3* operons, type II carries the *arc* operon but not *opp3*, type III contains *opp3* without *arc* operon. Type IV carries *arc* and *kdp* operons, while type V carries *arc*, *opp* and *kdp* operons [16, 19]. It has been suggested that ACME may also improve the fitness of CoNS and enhance their capacity to colonize human skin and mucosal surfaces [17]. In addition, other toxins such as staphylococcal enterotoxins and toxic shock syndrome toxin 1 (TSST-1), have has been reported in CoNS [20–22].

Studies from some European countries [23] and Brazil [24] have documented the contributions of CoNS in neonatal sepsis, which could result in increased risk of mortality and morbidities., There are limited data on the incidence of CoNS in neonatal sepsis in Kuwait [25, 26]. However, there are no studies on the molecular characteristics of CoNS associated with neonatal sepsis in Kuwait. Knowledge of the pathogenic characteristics of CoNS causing sepsis in neonates and their antibiotics susceptibility patterns will improve treatment outcomes and infection control measures. Consequently, CoNS obtained from blood cultures of neonates admitted to the Maternity hospital in Kuwait were investigated for antibiotic resistance, biofilm formation, and the carriage of other virulence determinants. In addition, the possible correlation between inflammatory response (C-reactive protein) and carriage of virulence factors were investigated.

## Materials and methods

### Setting

The Maternity Hospital in Kuwait is one of the tertiary care hospitals in the country. It has a capacity of approximately 465 beds. The rate of delivery is approximately 270 per month. The neonatal ward has two neonatal intensive care units (NICU) and two special care units with a capacity up to 160 incubators. The rate of admissions to the neonatal wards is approximately 327 per month.

### Data collection

This was a retrospective study conducted on 98 consecutive CoNS isolates which were collected from blood cultures of neonates at the Maternity hospital in Kuwait during six months between 1 August, 2015 and 31 January, 2016. The CoNS isolates used in this study were obtained as part of routine microbiology diagnostic investigations. The patient’s clinical characteristics (e.g. gestational age, birth weight, inflammatory markers, and presence of lines, diagnosis and treatment) were collected in the same period. Blood cultures were collected from a peripheral vein after skin cleansing with alcohol-based antiseptic. The diagnosis of sepsis caused by CoNS was made on clinical signs of sepsis in neonates with a positive monomicrobial blood culture and elevated C-reactive protein (CRP) (> 10 mg/L) within 2 days of blood culture positivity.

### Ethical approval

This study was approved by the Ethics Committee of the Medical Review Board, Ministry of Health, Kuwait. Approval No.2015/289. The approval was for the collection of patient’s clinical data. No consent was requested for the use of the isolates.

### Bacterial isolation and identification

Positive-Blood cultures by BD BACTEC™ instrument were sub-cultured on 5% sheep blood agar plate. Bacteria were isolated and identified to species level using semi-automated systems, VITEK 2 and VITEK MS (bioMérieux, France). Pure cultures of isolates on blood agar plates were submitted to the Gram-Positive Bacteria Research laboratory, located at the department of Microbiology, Faculty of Medicine, Kuwait University, where the isolates were retested for purity and preserved in 40% glycerol (v/v in brain heart infusion broth) at -80°C for further analysis. The isolates were recovered by two subcultures on brain heart infusion agar at 35°C before analysis.

### Antimicrobial susceptibility testing

Antimicrobial susceptibility testing was performed using disk diffusion on Mueller-Hinton agar according to Clinical Laboratory Standards Institute (CLSI) [27]. The following antibacterial agents were used (μg): penicillin G (10), fusidic acid (10), erythromycin (15), clindamycin (2), cefoxitin (30), gentamicin (10), rifampicin (5) and mupirocin (200). Minimum inhibitory concentration (MIC) of teicoplanin, vancomycin, cefoxitin, and mupirocin was determined by the Etest method (bioMérieux, France) and interpreted according to guidelines provided by CLSI. Methicillin resistance was confirmed by penicillin binding protein 2a (PBP2a) latex agglutination test according to the protocol provided by the manufacturer (OXOID, USA) [28], and the amplification of *mec*A [29] and *mec*C [30] genes.

### DNA extraction for PCR

All isolates stored at −80°C were thawed and grown *in vitro* on brain heart infusion agar (BHIA) **(BD Diagnostic systems, NJ)** plates and checked for strain purity prior to the DNA isolation. In the next step, 1–2 bacterial colonies were suspended in a lysing solution consisting of 50 μL lysostaphin (150 mg/ml) (Sigma-Aldrich Chemical Co., St. Louis, MO), 50 μL lysozyme (10 mg/ml) (Sigma-Aldrich Chemical Co., St. Louis, MO), and 10 μL RNase (10 mg/ml) (Sigma-Aldrich Chemical Co., St. Louis, MO) and incubated at 37°C for 40 minutes in a Thermoshaker (Eppendorf, Hamburg, Germany). Then, 50 μL of proteinase K (20 mg/ml) (Qiagen, Hilden, Germany) and 150 μL of tris buffer (0.1 M/ pH 8.0) were added and incubated at 60°C for 10 minutes in a water bath. The mixture was then incubated at 95°C for 10 minutes in Thermoshaker followed by brief centrifugation at 10,000 g for 30 seconds. The supernatant was used for PCR.

### Detection of *mecA* and *mecC* genes

All of the isolates were screened for *mecA* [29] and *mecC* [30] genes using primers and PCR protocols described previously. The PCR protocol for *mecA* gene start with 10 cycles consisting of initial denaturation at 94°C for 5 min followed by denaturation at 94°C for 45s, 65°C for 45s and 72°C for 1.5 min and followed by 25 cycles consisting of 94°C for 45s, 55°C for 45s, 72°C for 1.5 min and a final extension step at 72°C for 10 min. The PCR protocol for *mecC* gene consisted of 30 cycles including an initial denaturation step at 94°C for 15 min, followed by denaturation at 94°C for 30s, 59°C for 1 min. 72°C for 1 min and a final extension step at 72°C for 10 min. The PCR products were separated by 2.0% agarose gel electrophoresis and viewed under UV trans illumination.

### Detection of Staphylococcal Cassette Chromosome *mec (SCCmec)*

SCC*mec* typing was performed using multiplex PCR protocols according to protocols (the same PCR protocol used for *mecA* gene) published previously [29]. Detection of SCC*mec*-IV subtypes IVa, IVb, IVc, IVd, IVg and IVh was determined using multiplex PCR described by Zhang *et al*., [31] and Milheiriço *et al*., [32]. The PCR protocol for the SCC*mec* IV subtypes consisted of 35 cycles that start with an initial denaturation at 94°C for 4 min, followed by denaturation at 94°C for 30s, 48°C for 30s, 72°C for 2 min and a final extension step at 72°C for 4 min. Five μl of the PCR product was analysed in 2.0% agarose gel electrophoresis to confirm DNA amplification.

### Detection of genes for virulence factors

Clinical CoNS isolates were screened for the presence of virulence-associated genes including those for biofilm formation, *icaA*, *icaB*, *icaC and icaD* [33], Panton-Valentine leukocidin, *pvl* [34], staphylococcal enterotoxins, *sea*, *seb*, *sec*, *see*, *sed*, *seg*, *seh*, *sei*, and toxic shock syndrome toxin, *tst1* [35]. The distribution and type of ACME carried by the CoNS isolates was determined by detecting arginine catabolic mobile element *(*ACME) encoding genes *arc* and *opp3* [16]. ACME I contain both *arc* and *opp3*, ACME II contain only arc, while ACME III contain only *opp3*. The primers and references for amplification parameters for genes for the virulence factors are listed in Table 1. PCR products were separated by agarose gel electrophoresis using 2.0% agarose gel and visualized by UV trans illumination.

**Table 1:** Primer sequences for the amplification of genes encoding virulence factors.

Gene	Primer 5'-3'	Amplicon Size (bp)	Ref
***icaA***	F-GAC CTC GAA GTC AAT AGA GGT	814	Freeman *et al*., [27]
	R-CCC AGT ATA ACG TTG GAT ACC		
***icaB***	F-ATA AAC TTG AAT TAG TGT ATT	526	Freeman *et al*., [27]
	R-ATA TAT AAA ACT CTC TTA ACA		
***IcaC***	F-AGG CAA TAT CCA ACG GTA A	989	Freeman *et al*., [27]
	R-GTC ACG ACC TTT CTT ATA TT		
***icaD***	F-AGG CAA TAT CCA ACG GTA A	371	Freeman *et al*., [27]
	R-GTC ACG ACC TTT CTT ATA TT		
***arc***	F-GCAGCAGAATCTATTACTGAGCC	513	Zhang *et al*., [25]
	R-TGCTAACTTTTCTATTGCTTGAGC		
***opp-3***	F-GCAAATCTGTAAATGGTCTGTTC	1,183	Diep *et al*., [12]
	R-GAAGATTGGCAGCACAAAGTC		
***pvl***	F-CTGGTGCGATTCATGGT	433	Lina *et al*., [28]
	R-CGATATCGTGGTCATCA		
***tst***	F-ATCGTAAGCCCTTTGTTG	578	Udo *et al*., [29]
	R-GTGGATCCGTCATTCATTG		
***sea***	F-CATTGCCCTAACGTTGAC	619	Udo *et al*., [29]
	R-CGAAGGTTCTGTAGAAGTATGG		
***seb***	F-CTAAACCAGATGAGTTGCAC	489	Udo *et al*., [29]
	R-CCAAATAGTGACGAGTTAGG		
***sec***	F-GTAAAGTTACAGGTGGCAAAACTTG	296	Udo *et al*., [29]
	R-CATATCATACCAAAAAGTATTGCCGT		
***see***	F-GCTTTAAGCAATCTTAGGC	330	Udo *et al*., [29]
	R-CTATCCACAAGTTAATTGGTAC		
***sed***	F-GAATTAAGTAGTACCGCGCTAAATAATA	491	Udo *et al*., [29]
	R-GCTGTATTTTTCCTCCGAGAGT		
***seg***	F-TCTTTATACGTCTCCACC	326	Udo *et al*., [29]
	R-GTCTATTGTCGATTGTTACC		
***sei***	F-CAACTCGAATTTTCAACAGGTAC	466	Udo *et al*., [29]
	R-CAGGCAGTCCATCTCCTG		
***seh***	F-CGAAAGCAGAAGATTTACACG	341	Udo *et al*., [29]
	R-ACCAATCACCCTTTCCTGTG		

### Statistical analysis

Comparison between antibiotic resistance, biofilm formation, ACME, toxin production, and the immunological parameter, C-reactive protein (CRP) was done using Chi-square test, Mann-Whitney test and T-student test. P-value ≤ 0.05 was considered to be significant.

## Results

In total, 76 neonates were included in the study. Of those, 15 neonates had more than one septic episode during the study period. The neonates, 40 males and 36 females, were clinically unwell and were admitted to the Neonatal Intensive Care Units (NICU) or Special Care Units (SCU). The most common diagnosis was preterm birth observed in 66 neonates with a median gestational age of 29 weeks (24–41) and low birth weight with a median of 1,220 grams (480–4170 gm). Sixty-nine (90.7%) of the neonates had either central or peripheral lines. Sixty-seven (88.1%) neonates received antimicrobial treatment for sepsis due to bacterial or fungal infections.

Blood cultures from the 76 neonates yielded growth of 98 CoNS isolates. Ninety-three of the 98 yielded pure cultures while five cultures were mixed and were excluded from further analysis.

The 93 suspected septic episodes were classified as sepsis or non-sepsis group based on clinical presentation, CRP, time to positivity of blood culture (less than 24 hours), and the presence of a single organism in the blood culture. Using these criteria, 17 neonates belonged to the sepsis group and 59 neonates belonged to the non-sepsis group.

The Demographic data for the neonates are listed in Table 2. There were no significant differences between the two groups with regards to gestational age, birth weight, gender, diagnosis and treatment. However, CRP was significantly higher in the sepsis group (P = 0.00).

**Table 2:** Characteristic of neonates with coagulase-negative staphylococcal sepsis and non-sepsis blood cultures (n = 93).

Characteristics	Sepsis Group (n = 17)	Non sepsis Group (n = 76)	P-value
**Gestational age, mean wk**	30.76	30.15	0.633
Birth weight, mean g	1596	1491	0.684
**Gender, no (%)**			0.232
Male	11 (64.7)	37 (48.7)	
Female	6 (35.3)	39 (51.3)	
**Indwelling catheter, no (%)**			
Central	7 (41.2)	36 (47.4)	0.672
Peripheral	9 (52.9)	32 (42.1)	
None	1 (5.9)	8 (10.5)	
**CRP, mean mg/L**	38.25	12.18	0.00
Diagnosis*^1^**, no (%)**			0.904
PT, RDS	3 (17.6)	12 (15.8)	
PT, LBW	6 (35.3)	34 (44.7)	
Sepsis	3 (17.6)	10 (13.2)	
others	5 (29.4)	20 (26.3)	
**Treatment, no (%)**			0.084
Vancomycin	7 (41.2)	14 (18.7)	
Pipracillin/Tazobactum	6 (35.3)	9 (12.0)	
Cloxacillin & Amikacin	2 (11.8)	11 (14.7)	
Ampicillin & Amikacin	1 (5.9)	10 (13.3)	
Vancomycin & other	1 (5.9)	3 (4.0)	
antimicrobials			
Others*^2^	1 (5.9)	18 (24.0)	
No antibiotics	-	10 (13.3)	

* PT, RDS = preterm with respiratory distress, PT, LBW = preterm with low birth weight, others: intrauterine growth retardation, hypoglycemia, congenital anomalies, hydrocephalus, omphocele, necrotizing enter colitis, candidemia, diaphragmatic hernia.

Others*^2^: ceftriaxone, cefotaxime, meropenem, ambisome, amphotericin B, fluconazole, caspofungin.

### Identification of CoNS species

The majority of the 93 CoNS isolates were *S*. *epidermidis* (N *=* 76; 81.7%). This was followed by *S*. *capitis* (N = 12; 12.9%), *S*. *hominis* (N = 2; 2.1%), *S*. *warneri* (N = 2, 2.1%) and *S*. *haemolyticus* (N = 1; 1.0%).

### Antibiotic resistance of CoNS isolates

The prevalence of antibiotic resistance of the CoNS isolates is presented in Table 3. In total, 49 (52.7%) of the CoNS isolates consisting of 47 *S*. *epidermidis*, one *S*. *capitis* and one *S*. *hominis* expressed multiple resistance to antibiotics (were resistant to three or more classes of antibiotics). All isolates were sensitive to vancomycin (MIC: ≤ 2 μg/ml). However, three *S*. *epidermidis* isolates expressed intermediate resistance to teicoplanin (MIC: 16 μg/ml). The CoNS isolates where highly resistant to penicillin G, fusidic acid, gentamicin and cefoxitin. A total of 86 CoNS isolates comprising 69 (90.8%) of *S*. *epidermidis* and 17 (100%) non-*epidermidis* were positive for *mecA*. Five *S*. *epidermidis* isolates expressed phenotypic resistance to cefoxitin by MIC, yielded positive results for PBP2a but were negative for *mecA* and *mecC*. On the other hand, two *S*. *epidermidis* and one *S*. *haemolyticus* isolates yielded negative results for PBP2a but expressed phenotypic resistance to cefoxitin by MIC and were positive for *mecA*. *S*. *epidermidis* isolates were significantly more resistant to erythromycin (P = 0.00) and clindamycin (P = 0.07) than non- *epidermidis* isolates. None of the isolates was positive for *mecC*.

**Table 3:** Comparison of antibiotic resistance of *Staphylococcus epidermis* and non-epidermidis blood isolates obtained from neonates.

Antibiotic Resistance n, (%)	*Staphylococcus epidermis* (n = 76)	CoNS non-*epidermidis* (n = 17)	P-value
Penicillin	75 (98.7)	17 (100)	1.000
Fusidic acid	74 (97.4)	15 (88)	0.245
Erythromycin	45 (59.2)	2 (11.8)	0.000
Clindamycin	40 (52.6)	2 (11.8)	0.007
Gentamicin	59 (77.6)	13 (76.5)	0.938
Rifampicin	9 (11.8)	0 (0)	0.203
Mupirocin 200	6 (7.9)	2 (11.8)	0.635
Vancomycin MIC (32 μg/ml)	0 (0.0)	0 (0.0)	
Teicoplanin			
MIC (32 μg/ml) MIC	0 (0.0)	0 (0.0)	
(16 μg/ml)	3 (3.2)	0 (0.0)	0.45
Cefoxitin	53 (69.7)	0 (0.0)	0.096
*mecA* +ve	69 (90.8)	17 (100)	0.193

There were no significant differences in antibiotic resistance between the sepsis and non-sepsis groups.

### Distribution of SCC*mec* types

The 86 *mecA*-positive isolates were subjected to SCC*mec* typing to determine the distribution of SCC*mec* types among them. The dominant SCC*mec* types were types III and IVa, either alone or in combinations. In total, 33 MR-CoNS isolates including 22 *S*. *epidermidis* and 11 *S*. *capitis* carried SCC*mec* type III, while nine *S*. *epidermidis* isolates carried SCC*mec* type IVa. Four different combinations of SCC*mec* types were detected among the MR-CoNS isolates. SCC*mec* types I+IVa was detected in 25 *S*. *epidermidis* and one *S*. *warneri* isolates. Six *S*. *epidermidis* isolates carried SCC*mec* types III+IVa, while SCC*mec* type I+III and I+III+IVa were detected in single *S*. *epidermidis* isolate. SCC*mec* types could not be determined (un-typable) for 15 MR-CoNS isolates including *S*. *epidermidis* (N = 10), *S*. *hominis* (N = 2), *S*. *capitis* (N = 1), *S*. *warneri* (N = 1) and *S*. *hamolyticus* (N = 1).

### Biofilm formation by CoNS isolates

The 93 CoNS strains were tested for their ability to form biofilms by a genotypic method. In total, 57 (61.3%) of the isolates yielded positive results for genes encoding biofilm formation. The biofilm-forming genes, *icaA*, *icaB*, *icaC* and *icaD* were detected in 56 (73.6%) *S*. *epidermidis*. As shown in Table 4, *icaC*, detected in 54 (58.0%) isolates, was the most common biofilm encoding gene, followed by *icaA* detected in 52 (68.4%) isolates. *icaD*, was detected in 45 (59.2%) isolates and *icaB*, was detected in 42 (55.3%) isolates. Of the 76 *S*. *epidermidis* isolates, 38 (50%) isolates were positive for four genes, *icaA*, *icaB*, *icaC and icaD*, seven (7.5%) isolates were positive for three genes i*caA*, *icaC and icaD*, eight isolates (8.6%) were positive for two genes, *icaA and icaC* and three isolates (3.2%) were positive for either *icaA*, *icaB*, or *icaC*. Twenty *S*. *epidermidis* isolates were negative for all tested biofilm forming genes. Although the prevalence of biofilm formation genes was higher in the sepsis than in the non-sepsis group, the data presented in Table 4 showed no significant differences between sepsis and non-sepsis groups.

**Table 4:** Biofilm production and genetic determinant of virulence factors in 93 blood isolates of different CoNS species obtained from neonates (Sepsis group vs Non-sepsis group).

Virulence Factors n, (%)	Sepsis Group(n = 17)	Non-sepsis Group(n = 76)	P-value
**Biofilm encoding genes**			
*icaA*	12 (70.6)	40 (52.6)	0.178
*icaB*	9 (52.9)	33 (43.3)	0.476
*icaC*	12 (70.6)	42 (55.3)	0.247
*icaD*	11 (64.7)	34 (44.7)	0.136
**ACME genes**			
*arc*	7 (41.2)	27 (35.5)	0.662
*opp-3*	1 (5.9)	9 (11.8)	0.473
**Staphylococcal enterotoxin genes**			
*seb*	11 (61.8)	47 (61.8)	0.528
*sec*	1 (5.9)	7 (9.2)	0.550
*sed*	4 (23.5)	31 (40.8)	0.146
*seg*	0 (0.0)	15 (19.7)	0.036
*seh*	2 (11.8)	14 (18.4)	0.401
*sei*	9 (52.9)	26 (34.2)	0.123
**Toxic shock syndrome toxin genes**			
*tst*	1 (5.9)	1 (1.3)	0.241

### Detection of the arginine catabolic mobile element (ACME) in CoNS isolates

The distribution of the ACME genes, *arc* and *opp-3*, among the 93 CoNS isolates is summarized in Table 4. In total, *arc* was detected in 34 (38.2%) isolates consisting of 31 *S*. *epidermidis*, two *S*. *capitis* and one *S*. *haemolyticus* while *opp-3* detected in ten (13.1%) *S*. *epidermidis* isolates. Ten *S*. *epidermidis* isolates were positive for *arc* and *opp-3* and belonged to type I ACME, while 21 *S*. *epidermidis*, two *S*. *capitis* and the single *S*. *haemolyticus* were positive for *arc* only and belonged to type II ACME. None of the isolates belonged to type III ACME. There was no significant difference between sepsis and non-sepsis groups in the carriage of ACME genes although it was more common in the non-sepsis group (Table 4). However, 32 of the 33 *arc-* positive and 9 of the 10 *opp-3-* positive isolates were methicillin-resistant.

### Detection of toxin genes in CoNS isolates

**Fig 1:**
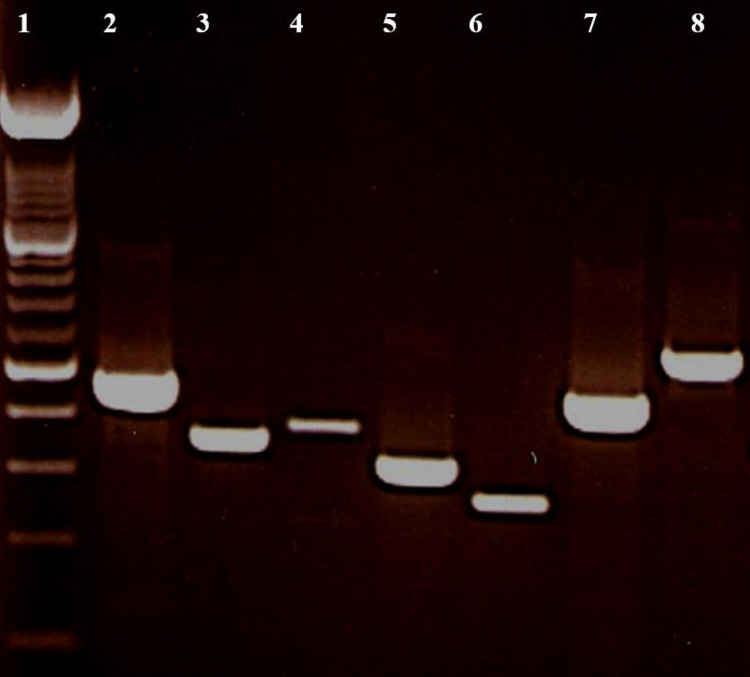
The amplified product of enterotoxins and TSST-1. Lane 1: 100 bp ladder; Lane 2: enterotoxin *seb*; Lane 3: enterotoxin *seh*; Lane 4: enterotoxin *sed*; Lane 5: enterotoxin seg; Lane 6: enterotoxin *sec;* Lane 7: enterotoxin *sei*; Lane 8: toxic shock toxin (*tst*) gene.

The 93 CoNS isolates were tested for the carriage of genes for Panton-Valentine leukocidin, *pvl*, staphylococcal enterotoxins, *sea*, *seb*, *sec*, *see*, *sed*, *seg*, *seh*, *sei* and toxic shock syndrome toxin, *tst1* (Fig 1). None of the 93 isolates was positive for *pvl*. However, the isolates were positive for different staphylococcal enterotoxins genes. The *seb* was the most prevalent SE gene and was detected in 46 *S*. *epidermidis*, eight *S*. *capitis*, two *S*. *hominis*, one *S*. *warneri*, and one *S*. *haemolyticus*. None of the isolates was positive for *sea*. Only two isolates, one *S*. *epidermidis* and one *S*. *warneri*, were positive for *tst1*.As shown in Tables 4, *seg* was more common in the non-sepsis group (P = 0.036) than the sepsis group. The isolates were positive for one to four SE genes. Most of the isolates were positive for two (N = 24) or three (N = 21) SE genes with *seb*, *sei* and *seb*, *sed*, *sei* detected in 7 and 12 isolates respectively. Four isolates were positive for four SE genes (Table 5).

**Table 5:** Prevalence of staphylococcal enterotoxins (SE) genes among CoNS isolates.

Genes for Staphylococcal Enterotoxins (SE)	No.
**Single SE genes**	
*Seb*	58
*Sed*	36
*sei*	35
*seh*	16
*seg*	15
*sec*	8
**Double SE genes**	
*seb*, *sei*	7
*sed*, *seg*	4
*sed*, *seh*	3
*seb*, *sed*	2
*seb*, *seh*	2
*seb*, *seg*	2
*sed*, *sei*	1
*seh*, *sei*	1
*seb*, *sec*	1
*sec*, *sei*	1
**Triple SE genes**	
*seb*, *sed*, *sei*	12
*seb*, *sec*, *sed*	2
*seb*, *seg*, *sei*	1
*sed*, *seh*, *sei*	1
*seb*, *sed*, *seg*	1
*sec*, *sed*, *sei*	1
*sed*, *seh*, *sei*	1
*sec*, *sed*, *seh*	1
*seb*, *sec*, *sei*	1
**Four SE genes**	
*seb*, *sed*, *seg*, *sei*	3
*sec*, *sed*, *seh*, *sei*	1

### Correlation between CRP, antibiotic resistance and virulence factors

There was no association between CRP values and gender, antibiotic resistance, biofilm formation, ACME or toxin production. Similarly, no association between biofilm formation and the presence of central or peripheral lines was found. However, biofilm formation was significantly associated with antibiotic resistance in *S*. *epidermidis* isolates. Biofilm- producing *S*. *epidermidis* isolates (N = 52; 98.1%) positive for *mecA* gene were more than biofilm-negative isolates (N = 17; 73.9%). In contrast, *arc* (gene for ACME) was significantly (P = 0.004) more common in biofilm-negative *S*. *epidermidis* isolates (N = 15; 65.2%).

## Discussion

This study investigated CoNS isolated from blood cultures of neonates for antibiotic resistance and the carriage of genes for virulence including genes for biofilm formation, staphylococcal enterotoxins, and ACME. Most of the neonates in this study were preterm, had low birth weight, and were clinically unwell which made them more susceptible to bacterial or fungal infections which mirrored the established facts that preterm infants are at greater risk of developing neonatal sepsis which may lead to increased length of stay in the ICU and higher mortality [1,6,36]. Therefore, provision of scientific data on the prevalence of antibiotic resistance and virulence characteristics of blood stream isolates of CoNS is important for better management and control of CoNS infections [7, 37].

*S*. *epidermidis* is widely reported as the most common cause of neonatal bloodstream infections [5,15,37]. Our results which showed that *S*. *epidermidis* constituted 81.7% of CoNS species is consistent with this notion and was similar to the results of previous studies in Norway [15], Brazil [38] and Turkey [39] where *S*. *epidermidis* was reported as the most common CoNS species associated with neonatal bloodstream infections.

The results showed that 49 (52.7%) of the CoNS isolates expressed multiresistance to antibiotics including resistance to penicillin G, fusidic acid, gentamicin, and cefoxitin. In addition, three *S*. *epidermidis* isolates expressed intermediate resistance to teicoplanin. Similarly, studies from different centres have also reported high prevalence of antibiotic resistance in *S*. *epidermidis* isolates [1, 15, 40, 41]. The results also showed that *S*. *epidermidis* isolates were more resistant to antibiotics than the non-*epidermidis* isolates. However, the differences were not significant except for resistance to erythromycin and clindamycin which were more prevalent among the *S*. *epidermidis* isolates. Surprisingly, despite being conducted several years apart, the prevalence of resistance to erythromycin and clindamycin in this study was similar to a previous study conducted in Mubarak Al-Kabeer Hospital, Kuwait in 1995 where 26.6% and 49.5% of the *S*. *epidermidis* were resistant to clindamycin and erythromycin respectively [40]. This highlights the importance of *S*. *epidermidis* as reservoirs for antibiotic resistance determinants. There was no significant difference between sepsis and non-sepsis groups of isolates with regards to antibiotic resistance (Table 3). This could be because the neonates were colonized with CoNS from the hospital environment which are usually resistant to antibiotics due to higher utilization of antibiotics in NICUs [42].

Our results also revealed discrepancies between the phenotypic and genotypic methods used for detecting methicillin resistance in CoNS species. Five *S*. *epidermidis* isolates were cefoxitin resistant and PBP2a positive but negative for *mecA* and *mecC* genes while two *S*. *epidermidis* and one *S*. *haemolyticus* were negative for *mecA* but were resistant by MIC which were similar to results reported previously in *S*. *epidermidis* isolates from adult patients [28]. The discrepant results could be due to the location of the *mecA* outside the coverage of the primers used in this study. These results make it imperative to adopt more than one method for susceptibility testing of CoNS isolates.

SCC*mec* typing of the CoNS isolates in this study revealed that they harboured single or combinations of SCC*mec* genetic elements. Most of the *S*. *epidermidis* carried SCC*mec* III as was also reported by Pinheiro *et al*., [43] in Brazil. The results also revealed that the MR-CoNS isolates carried different combination of SCC*mec* genetic elements which seems to be a common feature in MR-CoNS isolates [14].

The ability to form biofilm is an important virulence factor of *S*. *epidermidis* of clinical origin. The detection of biofilm forming genes in 61.3% of the CoNS in this study confirms the significance of those genes to the CoNS isolates. The higher positive rate for *ica* genes in *S*. *epidermidis* compared to the non-epidermidis observed in this study is similar to the findings by de Silva *et al*., [44] who also detected *ica* in more *S*. *epidermidis* than non-epidermidis. The detection of *ica* genes in more *S*. *epidermidis* isolates than non-*S*. *epidermidis* isolates could be because the primers for the detection of *ica* genes in both studies were based on the sequences of *S*. *epidermidis* genome [44]. However, whereas de Silva *et al*., [44] detected *icaC* and *icaD* in 41% of *S*. *capitis*, *icaC* was detected in only one *S*. *capitis* isolate in our study suggesting that these genes might be homologues of the *S*. *epidermidis ica* operon [44].

Two types of ACME were detected in *S*. *epidermidis*, *S*. *capitis* and *S*. *haemolyticus* in this study. Ten (10.7%) *S*. *epidermidis* isolates carried type I ACME whereas 21 *S*. *epidermidis*, two *S*. *capitis* and one *S*. *haemolyticus* harboured type II ACME indicating that type II ACME was more widespread among the CoNS isolates studied. The prevalence of ACME type I in this study was lower than the 24.8% prevalence of ACME type I in *S*. *epidermidis* reported by Barbier *et al*., [17]. In contrast, Granslo *et al*., [45] detected all three ACME types in *S*. *epidermidis* isolates that they studied suggesting differences in ACME carriage in *S*. *epidermidis* isolates in different geographical regions. In this study, there was no association between antibiotic resistance and the presence of ACME genes similar to the findings of other studies [45,46]. Surprisingly ACME positive isolates were more common among the non-sepsis group than the sepsis group. Similarly, Granslo *et al*., [45], found ACME positive *S*. *epidermidis* more abundant among the colonizing isolates.

The isolates in this study varied in the carriage of genes for staphylococcal enterotoxins. The detection of *seb* (62.3%) as the dominant SE gene in this study was similar to a study conducted by Udo *et al*., [20] on CoNS isolated from adult patients. In contrast, Vasconcelos *et al*., [47] detected *sei* as the most common SE among CoNS isolates obtained from newborns in Brazil [47]. These observations highlight the diversity in the carriage of SE genes in CoNS isolated in different geographical locations; thus strengthening the need to study these properties in each location. Our results also showed that most of the CoNS isolates were positive for multiple SE genes with 29% harbouring three or more SE genes. However, other studies conducted in other countries reported more CoNS with single SE genes [20, 48]. In a previous study conducted by Udo *et al*., [20], *tst1* was detected in 7% of the isolates which is more than the 2.1% prevalence detected in this study.

There was no association between CRP values and antibiotic resistance or biofilm formation in our study. In contrast, Klingenberg *et al*., [15] found higher CRP values in sepsis episodes with methicillin/aminoglycoside resistant versus methicillin/aminoglycoside susceptible CoNS. They also identified a significantly lower CRP response in neonates with CoNS sepsis caused by biofilm positive versus negative isolates. In this study, no association was found between ACME and CRP. Similarly, Granslo *et al*., [45] found no difference in the inflammatory response (CRP) in patients with ACME positive isolates compared with patients with ACME negative isolates.

This study has some limitations. Distinguishing between true CoNS bacteraemia and blood culture contamination in neonates is difficult. To determine neonatal CoNS sepsis in the scenario of only single positive blood culture requires the addition of clinical signs of sepsis and either at least 5 days of antibacterial therapy or elevated CRP values. These additional criteria all have limitations. CRP values may be elevated by non-infectious conditions. Still, we acknowledge that a proportion of the CoNS sepsis episodes may have represented contamination and vice versa, potentially skewing our comparisons.

In conclusion, *S*. *epidermidis* was the most common species isolated from neonatal blood culture at the Maternity hospital in Kuwait. The isolates were highly resistant to antibiotics with 49 (52.7%) being multidrug resistant. Most of the isolates were positive for genes encoding biofilm formation. ACME genes were detected in *S*. *epidermidis*, *S*. *capitis* and *S*. *haemolyticus*. Two types of ACME I and II were detected. Most of the isolates were positive for more than one enterotoxin gene and only two isolates were positive for *tst1*. All the isolates were negative for *pvl*. There was no association between immune response (CRP) and antibiotic resistance, biofilm formation, ACME or toxin production. This study has contributed to our understanding of antibiotic resistance and carriage of virulence determinants in CoNS causing neonatal bacteraemia.

## Supporting information

S1 Raw images (TIF)
