# Supplementary figures and images for: Antimicrobial resistance and virulence determinants in coagulase-negative staphylococci isolated mainly from preterm neonates
Source: PLoS One. 2020 Aug 4;15(8):e0236713. doi: 10.1371/journal.pone.0236713 (PMC7402503; doi:10.1371/journal.pone.0236713)

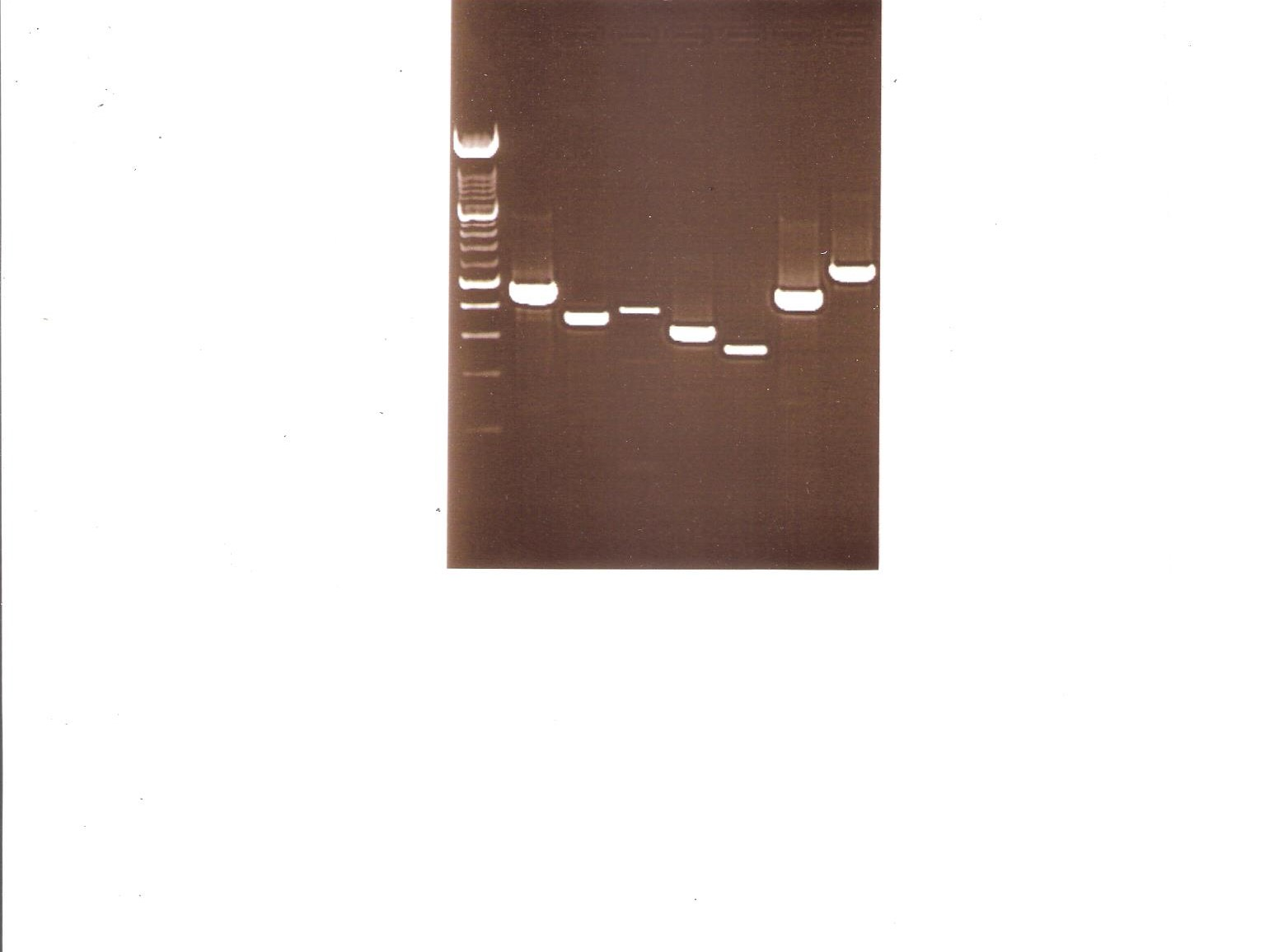

Supplement: S1 Raw images — (TIF) [file pone.0236713.s001.tif]
